# Factors Associated with Participation in Physical Activity Among Canadian School-Aged Children with Autism Spectrum Disorder: An Application of the International Classification of Functioning, Disability and Health

**DOI:** 10.3390/ijerph17165925

**Published:** 2020-08-14

**Authors:** Emily Bremer, Kathleen A. Martin Ginis, Rebecca L. Bassett-Gunter, Kelly P. Arbour-Nicitopoulos

**Affiliations:** 1 Faculty of Kinesiology and Physical Education, Mental Health and Physical Activity Research Centre, University of Toronto, Toronto, ON M5S 2W6, Canada; emily.bremer@utoronto.ca; 2 Department of Medicine, University of British Columbia, Vancouver, BC V6T 1Z3, Canada; kathleen_martin.ginis@ubc.ca; 3 School of Health & Exercise Sciences, University of British Columbia, Kelowna, BC V1V 1V7, Canada; 4 Centre for Chronic Disease Prevention and Management, University of British Columbia, Kelowna, BC V1V 1V7, Canada; 5 School of Kinesiology and Health Science, York University, Toronto, ON M3J 1P3, Canada; rgunter@yorku.ca

**Keywords:** ICF, disability, school, environmental factors, social factors

## Abstract

We have a limited understanding of the socioenvironmental factors associated with participation in physical activity among school-aged children with autism spectrum disorder (ASD), particularly regarding how the school environment may influence their participation. Using the International Classification of Functioning, Disability and Health (ICF) as a framework, this study examined the effect of body functions and structure, activity, and personal factors on in-school physical activity; and whether in-school physical activity, considered a socioenvironmental factor, is associated with out-of-school physical activity (i.e., participation) among elementary school-aged children (6–13 years of age) with ASD. Parents of 202 children with ASD (78.2% boys; M_age_ = 9.4 years) completed an online survey, as part of a larger study, to assess their child’s functioning and physical activity in- and out-of-school. Results indicated that the majority of children (85.1%) did not meet physical activity guidelines. In-school physical activities significantly predicted out-of-school physical activities including leisure-time moderate-to-vigorous physical activity (*R*^2^ = 0.27, *F*(10,154) = 5.67, *p* < 0.001) and meeting the physical activity guidelines (*R*^2^ = 0.23, *Χ*^2^ (10) = 31.9, *p* < 0.001). These findings underscore the importance of supporting children with ASD to be physically active in school, which may impact physical activity levels out-of-school.

## 1. Introduction

Autism spectrum disorder (ASD) is a neurodevelopmental disability that affects approximately 1.5% of North American children [1,2]. ASD is characterized by difficulties in the areas of social interactions and social communication, along with the presence of restricted or repetitive interests and behaviours [3]. In addition to the core difficulties experienced by children with ASD, this is a population that often experiences a high prevalence of medical, psychiatric, and developmental comorbidities [4]. Moreover, individuals with ASD can experience several adverse physical health outcomes including an increased risk of being overweight or obese [5,6] along with an increased risk of all-cause mortality [7].

Regular participation in physical activity has known benefits for a range of physical and mental health outcomes [8,9,10] and is increasingly recognized as a beneficial health and behavioural intervention for children with ASD [11,12,13]. Yet, previous research suggests that children with ASD engage in less physical activity overall [14,15], fewer types of physical activities [16], and are less likely to meet physical activity guidelines [17] than their neurotypical peers. We do not, however, have a comprehensive understanding of the socioenvironmental and personal factors that may be related to participation in physical activity among children with ASD. The World Health Organization’s International Classification of Functioning, Disability, and Health (ICF; [18]) provides one framework of disability and functioning that can be applied to our understanding of the factors associated with participation in physical activity among children with ASD. In short, the ICF views functioning, classified as body functions and structure, activity, and participation, as the outcome of interactions between a health condition and contextual factors [18]. As such, when considering physical activity as the desired participation outcome, it is important to consider one’s health condition (e.g., ASD), body functions and structure (e.g., the presence of any comorbidities), and activity (defined as the ability to execute given tasks or actions; e.g., functional abilities). However, we must also consider how contextual factors including socioenvironmental (e.g., physical or social environment) and personal (e.g., age) factors influence one’s participation. Understanding the interactions between these factors, as they relate to participation in physical activity among children with ASD, may help researchers and practitioners in effectively promoting physical activity among this population.

Research regarding the contextual factors related to participation in physical activity among children with ASD is limited. Recent reviews by Jones et al. [15] and Healy et al. [19], respectively, have identified a significant negative association between physical activity and age and significant positive associations between physical activity and male sex, but have not found meaningful associations between physical activity and additional personal factors, such as socioeconomic status or level of enjoyment in physical activity. The consideration of socioenvironmental factors on participation in physical activity among children with ASD may be even more critical given the ability to change aspects of one’s environment through intervention and policy. Yet, there is limited research on socioenvironmental factors and the limited research has yet to identify factors that are associated with participation in physical activity among children with ASD [15,19].

Schools are one environment that may be particularly important in developing children’s interest and participation in physical activity. Indeed, children spend a great deal of their time in school settings which position schools as an ideal setting for physical activity interventions [20]. One approach to targeting physical activity in school is through the framework of a comprehensive school physical activity program [21]. This framework has five guiding principles to enable students to meet physical activity guidelines, through school physical activity, each day. These principles include quality physical education, physical activity before and after school, physical activity across the school day (e.g., classroom activity breaks, lunch, and recess), staff involvement, and community engagement [22]. Comprehensive school physical activity programs have demonstrated success in increasing children’s in-school physical activity overall including time spent in moderate-to-vigorous physical activity (MVPA) during physical education, lunch, recess, and classroom time [23]. There is limited research specifically examining in-school physical activity among children with ASD; however, previous work suggests that these children are less active at recess [24] and during physical education [25,26] than their neurotypical peers. Given that the physical activity levels of children with ASD are generally quite low, it is important to understand how the school environment and personal factors may influence participation in physical activity.

**Figure 1:**
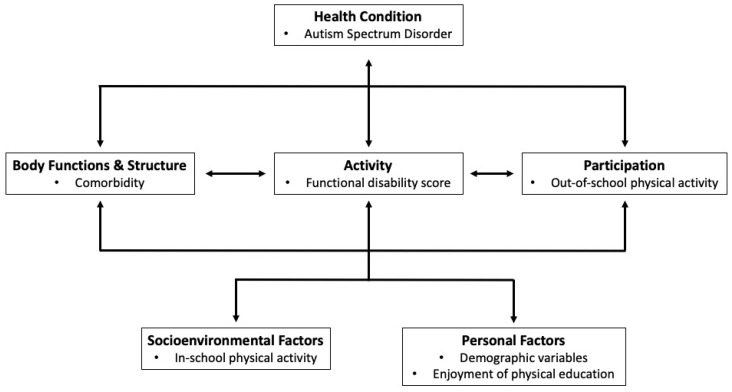
An adapted International Classification of Functioning, Disability and Health (ICF) model used to explore the associations between in- and out-of-school physical activity among school-aged children with autism spectrum disorder (ASD) (adapted to show the operationalization of the ICF constructs in this study).

While the evidence regarding the association between in-school physical activity on out-of-school physical activity has shown mixed results in neurotypical children [27,28,29,30], this relationship has not been explored among children with ASD. Understanding this relationship, along with rates of participation in in-school physical activity, among children with ASD can advance an understanding of the socioenvironmental factors related to out-of-school physical activity. Such knowledge can be used to effectively design physical activity programs that can capitalize on current patterns of participation and the influence of socioenvironmental and personal factors, while filling gaps in school services. Positioned within the ICF, the purpose of this study was twofold: first, to examine the effect of body functions and structure, activity (i.e., functional disability level), and personal factors on in-school physical activity; and second, to assess whether in-school physical activity (i.e., a socioenvironmental factor) is associated with out-of-school physical activity (i.e., participation) among elementary school-aged children with ASD. An adapted version of the ICF model with the variables explored in this study is presented in Figure 1.

## 2. Materials and Methods

### 2.1. Study Sample and Data Collection

Data for the current study originated from a larger study of Canadian children and youth with disabilities, the National Physical Activity Measurement (NPAM) study. The NPAM study is an on-going cross-sectional study of various movement behaviours among Canadian school-aged children and youth with physical, sensory, and developmental disabilities, 4 to 17 years of age. Participants in the NPAM study were recruited through a nationally organized sport and recreation-based charitable organization, as well as other community-based organizations and programs for children and youth with disabilities. The present study focused on a subsample of participants from the larger NPAM study. Eligibility criteria for this study included: a) being a parent of a child diagnosed with ASD who is elementary school-aged (i.e., between 6 and 13 years of age); b) living in Canada; c) being proficient in English (although the NPAM survey has since been expanded to French). The NPAM study was approved by the research ethics boards at the University of Toronto (#31862), University of British Columbia (#H17-02514), and York University (#e2015-328) and informed consent was obtained online from each participant prior to data collection. Data collection involved the one-time completion of an online survey that took parents approximately 30 min to complete.

### 2.2. Measures

Parents answered a series of questions regarding demographic information about themselves and their child, as well as their child’s functioning and physical activity behaviours. Physical activity questions were taken from four previously validated population-based surveys to provide a comprehensive picture of children’s in-school and out-of-school physical activity. Information regarding the outcome variables, organized by the five domains of the ICF (see Figure 1), is described below.

#### 2.2.1. Body Functions and Structure

Comorbid diagnoses. Parents indicated whether their child had additional diagnoses (e.g., diabetes, anxiety) beyond ASD. This outcome was recorded as a “yes” or “no” response.

#### 2.2.2. Activity

Functional disability score. Parents reported the severity of their child’s disability using five items from the Washington Group Short Set of Questions on Disability [31]. Parents reported their child’s difficulties across five domains including vision, hearing, mobility, cognition, and self-care. Each question was answered on a four-point scale from “no difficulty” to “unable to do”. Scores from the five items were used to calculate a functional disability score that ranges from 0 (no difficulties) to 1 (severe difficulties) [31].

#### 2.2.3. Participation

Out-of-school physical activity or sports participation. Two items from the National Longitudinal Survey of Children and Youth [32] were used to assess the frequency of the child’s out-of-school physical activity or sports participation, (a) with or (b) without a coach or instructor. These two items were prefaced with, “Outside of school, during the past 12 months, how often has your child…”. Responses were made using a 1 (never) to 4 (≥ 4 times per week) rating scale. An example of an activity with a coach would include swimming lessons, while an example of an activity without a coach would include a drop-in basketball game at a local recreation center. The two outcome variables from this measure included out-of-school participation in physical activity or sport with a coach and without a coach, respectively.

Leisure-time MVPA. The International Physical Activity Questionnaire for Adolescents (IPAQ-A; [33]) was used to assess the frequency and duration of the child’s physical activity behaviour in the last seven days in the domain of leisure-time physical activity (LTPA). In this context, LTPA included activities completed solely for recreation, sport, exercise, or leisure (i.e., excluding activities for therapy or transportation). Parents were asked not to include any activities that were already accounted for in the previous questions, such as activities done with or without a coach or in-school. Items from the IPAQ-A were modified such that participants were asked to recall their child’s total minutes of activity, irrespective of being in bouts of 10 or more minutes to better align with the 24-Hour Movement Guidelines [34] and ensure that incidental activity was captured. Parents were asked about their child’s moderate (i.e., activities that make them breathe somewhat harder than normal; e.g., dancing, swimming at a regular pace) and vigorous (i.e., activities that make them breathe much harder than normal; e.g., running, bicycling, fast-swimming) activity. The responses were then combined into a single outcome variable from this measure to indicate the time spent in leisure-time MVPA, recorded as minutes per week.

Meeting physical activity guidelines. One item from the Health Behaviour in School Children Survey (HBSC; [35] was used to assess whether children met the physical activity guidelines. Parents indicated the number of days in the previous week that their child participated in physical activity for at least 60 min per day. Children were then classified as meeting physical activity guidelines if they had engaged in at least 60 min per day of physical activity on each of the 7 days in the previous week. This item has previously demonstrated acceptable test-retest reliability and criteria-related concurrent validity among neurotypical children [36]. The one outcome variable from this measure was whether physical activity guidelines were met, recorded as a “yes” or “no”.

#### 2.2.4. Personal Factors

Demographics. Parents completed a demographic survey that asked about their level of education, employment status, household income, and geographical location. The survey also asked parents to report their child’s age, gender, and ethnicity. All of these variables were reported on a nominal scale except for education and household income, which were ordinal. The specific demographic items within the questionnaire are reported in Table 1.

Enjoyment of physical education. Parents completed one item from the Physical Activity Monitor (PAM) survey [37] to assess their perception of their child’s enjoyment of their physical education classes. The item-response scale ranged from 1 (absolutely does not enjoy) to 10 (absolutely enjoys) and the single outcome variable from this measure was the enjoyment of physical education.

#### 2.2.5. Socioenvironmental Factors

In-school physical activity or sports participation. Recognizing the potentially important social influence of coaches on children’s physical activity, we incorporated separate items assessing in-school physical activity with and without a coach. Specifically, two items from the National Longitudinal Survey of Children and Youth [32] were used to assess the frequency of the child’s in-school physical activity or sports participation, (a) with or (b) without a coach or instructor. These two items were prefaced with, “Since the beginning of the school year, how often has your child taken part in the following activities at school, other than during physical education class”. Responses were made using a 1 (never) to 4 (≥ 4 times per week) rating scale. An example of an activity with a coach would include a school extramural sports team (e.g., school soccer team), while an example of an activity without a coach would include an intramural league (e.g., lunchtime dodgeball league) or school-wide physical activity day (e.g., track and field day). The two outcome variables from this measure included in-school participation in physical activity or sport with a coach and without a coach, respectively.

Physical education time and recess MVPA. The IPAQ-A [33] was used to assess the frequency and duration of the child’s physical activity behaviour in the last seven days in the domains of physical education class and recess. Items from the IPAQ-A were modified such that participants were asked to recall their child’s total minutes of activity, irrespective of being in bouts of 10 or more minutes to better align with the 24-h Movement Guidelines [34] and ensure that incidental activity was captured. For physical activity at recess, parents were asked about their child’s moderate (i.e., activities that make them breathe somewhat harder than normal; e.g., dancing) and vigorous (i.e., activities that make them breathe much harder than normal; e.g., running) activity. The responses were then combined into a single outcome variable from this measure to indicate the time spent in MVPA at recess, recorded as minutes per week. Time spent in physical education was reported as an absolute value, rather than by intensity, and was also recorded in minutes per week.

### 2.3. Analyses

Descriptive statistics were calculated for all variables. To answer the first research question, chi-squared tests were used to assess whether children’s participation in in-school physical activity or sports programs differed across the measured body functions and structure, activity, or personal factors. Similarly, Kruskal–Wallis tests were used to assess whether time spent in physical education differed across the same variables.

To answer the second research question, Spearman correlations were first used to examine the relationships between in-school (i.e., socioenvironmental factors) and out-of-school physical activity (i.e., participation) variables. Spearman correlations were used given that in-school and out-of-school physical activity and sports with and without a coach were measured on ordinal scales. Four separate regression models were then tested to examine the effect of all in-school physical activity variables (in-school physical activity with and without a coach, physical education enjoyment and time, and recess MVPA) on out-of-school physical activity. First, linear regression was used to test whether in-school physical activity variables predicted out-of-school leisure-time MVPA, which was measured as a continuous variable. Second, two ordinal logistic regressions were used to test whether in-school physical activity predicted out-of-school physical activity with and without a coach, respectively, given that these outcomes were measured on an ordinal scale. Lastly, a binomial logistic regression was used to test whether in-school physical activity predicted whether participants were meeting physical activity guidelines, measured on a dichotomous scale. The functional disability score was entered as a covariate in each of the four regression models. All analyses were completed in Jamovi version 1.2.17 [38].

## 3. Results

### 3.1. Participant Characteristics

The study included 202 parents of children (78.2% boys) between the ages of 6–13 years (M_age_ = 9.4 years). Children were predominately Caucasian (72.3%) and living in the province of British Columbia, Canada (70.3%). The demographic characteristics of the children and their parents are presented in Table 1. Both in- and out-of-school physical activity levels were generally low. Just under half (44.3%) of the children had never participated in in-school physical activity or sports without a coach and slightly over one-third (36.3%) of children had never participated in these activities with a coach, respectively. There was a large amount of variability in total leisure-time MVPA with children averaging 157 ± 160 min per week. Moreover, the majority of children (85.1%) did not meet physical activity guidelines. Descriptive statistics of all physical activity outcomes are presented in Table 2.

### 3.2. Differences in In-School Physical Activity (Socioenvironmental Factors) by Body Functions and Structure, Activity, and Personal Factors

In-school physical activity did not vary by any of the measured body functions and structure or personal factor variables (all *p* values > 0.05). However, in-school physical activity without a coach was significantly related to the functional disability score (ρ = −0.167, *p* < 0.05); children experiencing greater levels of functional challenges had lower levels of participation.

### 3.3. Relationship Between In-School (Socioenvironmental Factors) and Out-of-School Physical Activity (Participation)

Significant positive correlations, that were small-to-medium in size (ρ values ranging from 0.16–0.57), were found between in-school physical activities and out-of-school physical activity participation (see Table 3).

The results of the regression analyses are presented in Table 4. In-school physical activities significantly predicted out-of-school leisure-time MVPA (*R*^2^ = 0.27, *F* (10,154) = 5.67, *p* < 0.001), out-of-school physical activity or sports without a coach (*R*^2^ = 0.20, *Χ*^2^ (10) = 85.6, *p* < 0.001), out-of-school physical activity or sports with a coach (*R*^2^ = 0.08, *Χ*^2^ (10) = 30.0, *p* < 0.001), and whether physical activity guidelines were met (*R*^2^ = 0.23, *Χ*^2^ (10) = 31.9, *p* < 0.001). Looking at the individual models, we see that participating in in-school physical activity or sports at least once a week and recess MVPA were significant independent predictors of out-of-school leisure time MVPA.

Out-of-school physical activity or sports without a coach was predicted by in-school physical activity or sports without a coach, as well as the enjoyment of physical education classes and functional disability scores. Specifically, participants engaging in in-school physical activity or sports without a coach, less than once per week, were 6.7 times more likely to have higher out-of-school physical activity or sports engagement without a coach compared to participants who never engaged in in-school physical activity or sports without a coach. These odds increased with more frequent participation: participants engaging in in-school physical activity or sports without a coach more than four times per week were 20.3 times more likely to have higher out-of-school physical activity or sports engagement without a coach compared to participants who never engaged in these activities in-school. The results also indicate that participants with a higher functional disability score (i.e., experiencing greater challenges) were less likely to engage in out-of-school physical activity or sports without a coach.

Out-of-school physical activity or sports with a coach was predicted by in-school physical activity or sports with a coach, as well as the enjoyment of physical education classes and functional disability scores. Specifically, we see that participants engaging in in-school physical activity or sports with a coach one to three times per week were 2.6 times more likely to have higher out-of-school physical activity or sports engagement with a coach compared to participants who never engaged in in-school physical activity or sports with a coach. These odds increase with more frequent participation: participants engaging in in-school physical activity or sports with a coach more than four times per week were 6.6 times more likely to have higher out-of-school physical activity or sports engagement with a coach compared to participants who never engaged in these activities in school. Similar to activities without a coach, the results also indicate that participants with a higher functional disability score (i.e., experiencing greater challenges) were less likely to engage in out-of-school physical activity or sports with a coach.

Lastly, participating in in-school physical activity or sports without a coach four times a week or more was the only significant independent predictor of meeting the physical activity guidelines, with participants who engaged in in-school physical activity or sports without a coach four times a week or more being 6.1 times more likely to meet the physical activity guidelines than participations who never engaged in in-school physical activity or sports without a coach.

## 4. Discussion

This is the first study to examine factors associated with in-school physical activity among Canadian school-aged children with ASD, within the context of the ICF, and whether children’s in-school physical activity is related to out-of-school physical activity participation. In-school physical activity without a coach was negatively related to children’s functional disability scores. However, we found that in-school participation in physical activity with or without a coach does not vary across a range of personal factors or measures of body functions and structure. We view this null finding positively, as it suggests that participation in in-school physical activity is generally not affected by factors that are commonly related to engagement in physical activity outside of school, such as age, gender, and socioeconomic status [39,40]. It is possible that Canadian schools can provide an environment for physical activity that is relatively free from the influence of these personal factors. Indeed, school is commonly situated as an ideal environment in which to intervene on physical activity among children in general [20], and it would appear that this may also be true among children with ASD in particular.

A second finding from this study was that in-school physical activities, particularly those done without a coach (e.g., school fun-runs), were related to out-of-school leisure-time MVPA, physical activity and sport participation, and meeting the physical activity guidelines. This finding is consistent with the comprehensive school physical activity framework which outlines the need to support children’s physical activity throughout the entire school day [21] and in doing so, we can increase physical activity both in- and out-of-school [23,41]. It is possible that opportunities to be active in school can help children with ASD to develop the skills, confidence, and enjoyment to be active outside of school. Further, these in-school physical activities contribute to overall physical activity levels and may help children with ASD to meet physical activity guidelines. It is not surprising that our results showed that children who engaged in in-school physical activity or sports programs without a coach four times a week or more were more likely to meet physical activity guidelines than those children not participating or participating to a lesser extent. Considering that the majority of children in our sample were not meeting physical activity guidelines, this finding underscores the need to engage children with ASD in in-school physical activity and sports programming, daily, through comprehensive school physical activity programs.

Beyond simply providing opportunities for children with ASD to be physically active in school, these in-school physical activity experiences must be enjoyable and of high quality. For example, while we did not find an association between time spent in physical education classes and out-of-school physical activity, we did find that parent’s perceptions of their child’s enjoyment of physical education (a personal factor) was a significant independent positive predictor of out-of-school physical activity and sports with and without a coach. This finding may be attributed to the fact that although the total time spent in physical education may vary between classrooms, schools, and provinces due to curriculum mandates and scheduling, there can be great variability between the experiences of children in physical education. Though the realization of inclusive physical education has proven difficult [42], where quality and effective inclusive physical education is not practiced, it is unlikely students with disabilities will fully benefit from in-school physical activity opportunities. Teachers play a critical role in realizing educational pedagogy, such as inclusive physical education, and as such, strategies that support teachers’ motivation to implement inclusive physical education should be further explored [43]. Previous research has highlighted that children with disabilities, including those with ASD, often have negative experiences in physical education that include negative peer interactions, forced exclusion, and self-exclusion from activities [44,45,46]. These negative experiences may be prohibitive to future, or out-of-school participation in physical activity resulting in a cyclical process of withdrawal and negative experiences. On the contrary, children with ASD who have enjoyable experiences in physical education may be more likely to engage in other physical activities and be provided with further skill-building experiences that are necessary for continued participation. It is important to acknowledge that our design precludes inferences about directionality. The relationship may act in the opposite direction: parents who see their child enjoying being active out-of-school may be more likely to report higher levels of their child’s enjoyment in physical education. Regardless of directionality, the importance of enjoyment is consistent with the framework of physical literacy, which positions enjoyment (along with competence, motivation, and knowledge) as one of the core components to being physically active for life [47,48]. Physical literacy is also considered the basis of a quality physical education program [49], further emphasizing the importance of providing positive, enjoyable experiences for all children in physical education.

While we did not measure the actual quality of children’s physical education classes, their parents’ perceptions of their enjoyment provide an important indicator of quality physical education [49,50]. The quality of physical education and comprehensive school physical activity programs may be even more important for children with ASD as the school setting can provide an opportunity to build the foundational skills needed to be active while making a substantial contribution to overall physical activity levels. However, providing these opportunities can pose a challenge to educators who may not be trained in providing quality physical activity/physical education programs, let alone adapted physical activity programming. In Canada, where this study was conducted, certified adapted physical education teachers are rare and many elementary schools do not even have specialist physical education teachers, rather they rely on generalist teachers to lead physical education [51]. Generalist teachers often do not possess the foundational knowledge needed to deliver physical education [52,53] and report lower levels of self-efficacy toward delivering physical education compared to their specialist peers [54]. This lack of knowledge and lower self-efficacy is likely exasperated when working with children with disabilities, including those with ASD, given their increased support needs [55,56]. Not surprisingly, we found that children’s functional disability score was negatively related to in-school physical activity without a coach, indicating that those children experiencing greater levels of functional challenges had lower levels of participation in these informal school activities. This finding is likely indicative of the school system being ill-equipped to support children with more complex needs, in general, let alone in the context of physical activity [57]. Children with ASD who participate in adapted physical education, rather than generalist physical education, typically report positive experiences [58,59]; reinforcing the need to provide schools and teachers with the training and support necessary to positively engage children with ASD in quality physical education and school physical activity programs. Beyond just enjoyment, individuals need to feel a sense of autonomy, belonging, challenge, engagement, mastery, and meaning to support their quality participation in physical activity [60]. Thus, we must continue to explore the experiences of children with ASD in in-school physical activities to understand their experiences and the quality of their participation to implement physical activity programs that lead to quality participation for children with ASD.

Although this study has provided new information regarding the influence of the school environment on participation in out-of-school physical activity among children with ASD, it is not without its limitations. First, children in our sample were predominately identified as boys, from one province, and households with relatively high socioeconomic status. Although we explored the influence of gender in this study, the rate of ASD diagnosis differs by sex with about four times more male children diagnosed than female children [2]. Moving forward, we must seek to understand how both sex and gender influence participation in physical activity. The limited diversity in geographical location and socioeconomic status of our sample may have also influenced our findings, particularly in regard to the lack of effect of personal factors on in-school participation in physical activities. It is possible that a more diverse sample, particularly in regard to socioeconomic status, would change the results. Second, we did not collect data on additional socioenvironmental factors that may influence school physical activity, such as the type of school attended (e.g., publicly vs. privately funded), school neighbourhood, or school programs offered and teacher training. Third, the cross-sectional design of this study does not allow us to infer the directionality of the associations between in- and out-of-school physical activities. Finally, the study was limited by parent-report, rather than surveys of the children themselves and objective measures of physical activity. In our experience working with families who have a child with a disability, parents tend to have a good grasp on their child’s daily activities as they are actively involved in virtually all aspects of their child’s life; however, future research should also explore these questions through child-reported measures. In addition, future research should continue to examine personal and socioenvironmental factors related to in-school physical activity, along with its association to participation in physical activity out-of-school through both experimental and longitudinal research designs that employ mixed-methods approaches to further disentangle the physical activity experiences of children with ASD in relation to their participation.

## 5. Conclusions

In conclusion, the results of this study highlight the importance of in-school physical activity as an important socioenvironmental factor associated with participation in physical activity among Canadian school-aged children with ASD. Participation in in-school physical activity does not appear to vary across a range of personal factors and it is positively associated with out-of-school physical activity including leisure-time MVPA, activities with and without a coach, and meeting physical activity guidelines. Future work should continue to explore the role of in-school physical activity, with an emphasis on creating comprehensive school physical activity programs that are of high quality, enjoyable, and accessible for children with ASD.

## Figures and Tables

**Table 1:** Descriptive characteristics of child- and parent-level variables.

Participant	Measure	Mean (SD), IQR or N, %
Child	Age	9.4 (2.1), 3
	Gender	
	Boy	158, 78.2%
	Girl	41, 20.3%
	Transgender	2, 1.0%
	Prefer not to answer	1, 0.5%
	Comorbidity	
	No	155, 76.7%
	Yes	47, 23.3%
	Functional Disability Score	0.2 (0.1), 0.1
	Ethnicity	
	Asian (East, South, and Southeast)	19, 9.4%
	Black	4, 2.0%
	Caucasian	146, 72.3%
	Hispanic	1, 0.4%
	Indigenous	9, 4.5%
	Mixed Ethnicity	21, 10.4%
	Prefer not to answer	2, 1.0%
Parent	Province of Residency	
	British Columbia	142, 70.3%
	Alberta	16, 7.9%
	Ontario	36, 17.8%
	Other	8, 4%
	Education Level	
	Less than College diploma	52, 25.9%
	College diploma	60, 29.9%
	Bachelor’s degree	72, 35.8%
	Graduate or Professional degree	17, 8.4%
	Employment Status	
	Employed	152, 75.2%
	Unemployed	40, 19.8%
	Student	5, 2.5%
	Prefer not to answer	5, 2.5%
	Household Income	
	Less than $50,000	48, 27.3%
	$50,000 to $99,999	67, 38%
	Over $100,000	61, 34.7%

Note: IQR = Interquartile Range.

**Table 2:** Participation in in-school and out-of-school physical activity.

Setting	Outcome	Mean (SD), IQR or N, %
In-School	Physical activity or sports, without a coach	
	Never	89, 44.3%
	<1 time per week	46, 22.9%
	1–3 times per week	39, 19.4%
	≥4 times per week	27, 13.4%
	Physical activity or sports, with a coach	
	Never	73, 36.3%
	<1 time per week	39, 19.4%
	1–3 times per week	72, 35.8%
	≥4 times per week	17, 8.5%
	Physical education time (min/week)	120 (87.9), 113
	Physical education enjoyment	6.5 (2.6), 3
	Recess MVPA (min/week)	85.8 (92.1), 150
Out-of-School	Physical activity or sports, without a coach	
	Never	57, 28.4%
	<1 time per week	67, 33.3%
	1–3 times per week	57, 28.4%
	≥4 times per week	20, 10%
	Physical activity or sports, with a coach	
	Never	28, 13.9%
	<1 time per week	44, 21.9%
	1–3 times per week	113, 56.2%
	≥4 times per week	16, 8%
	Leisure-time MVPA (min/week)	157 (160), 213
	PA guidelines met	
	Yes	30, 14.9%
	No	172, 85.1%

Note: IQR = Interquartile Range; MVPA = Moderate-to-Vigorous Physical Activity; PA = Physical Activity.

**Table 3:** Spearman correlations between in-school and out-of-school physical activity.

Environment		In-School				
	Variable	Physical Activity or Sports, without a Coach	Physical Activity or Sports, with a Coach	Physical Education (min/week)	Physical Education Enjoyment	Recess MVPA (min/wk)
Out-of-School	Physical activity or sports, without a coach	0.57 ***	0.16 *	−0.09	0.22 **	0.19 **
	Physical activity or sports, with a coach	0.11	0.29 ***	−0.04	0.21 **	0.13
	Leisure-time MVPA (min/wk)	0.34 ***	0.24 ***	0.12	0.30 ***	0.37 ***

Note: * *p* < 0.05; ** *p* < 0.01; *** *p* < 0.001; MVPA = Moderate-to-Vigorous Physical Activity.

**Table 4:** In-school physical activity as a predictor of out-of-school physical activity and meeting the physical activity guidelines.

Outcomes	Out-of-School Leisure-Time MVPA		Out-of-School PA/Sports, without a Coach	Out-of-School PA/Sports, with a Coach	PA Guidelines Met
Predictors	*b* (SE)	Stand. β (95% CI)	Odds Ratio (95% CI)	Odds Ratio (95% CI)	Odds Ratio (95% CI)
School PA/sports, without a coach					
<1 / week	−0.7 (29.1)	−0.004 (−0.4–0.4)	6.7 (2.9–15.9) ***	1.7 (0.7–3.9)	0.2 (0.03–2.3)
1–3 / week	89.0 (30.5) **	0.6 (0.2–1.0)	14.2 (5.7–36.9) ***	1.0 (0.4–2.3)	0.9 (0.2–4.0)
≥4 / week	113.8 (32.9) ***	0.7 (0.3–1.2)	20.3 (7.4–58.3) ***	0.9 (0.4–2.4)	6.1 (1.7–21.9) **
School PA/sports, with a coach					
<1 / week	35.3 (31.0)	0.2 (−0.2– 0.6)	1.1 (0.5–2.6)	1.0 (0.5–2.5)	0.9 (0.2–4.9)
1–3 / week	29.1 (26.0)	0.2 (−0.1–0.5)	1.5 (0.7–3.0)	2.6 (1.2–5.5) *	1.1 (0.3–3.7)
≥4 / week	50.2 (39.4)	0.3 (−0.2–0.8)	1.5 (0.5–4.4)	6.6 (2.0–23.3) **	4.2 (0.8–20.5)
PE enjoyment	4.0 (4.5)	0.1 (−0.1–0.2)	1.2 (1.0–1.3) *	1.2 (1.0–1.4) **	1.2 (1.0–1.5)
PE min / week	0.2 (0.1)	0.1 (−0.01–0.3)	0.9 (0.9–1.0)	0.9 (0.9–1.0)	1.0 (0.9–1.0)
Recess MVPA	0.3 (0.1) **	0.2 (0.1–0.4)	1.0 (0.9–1.0)	0.9 (0.9–1.0)	1.0 (0.9–1.0)
Functional Disability	−126.7 (103.0)	−0.1 (−0.2–0.1)	0.04 (0.002–0.6) *	0.05 (0.002–0.9) *	88.3 (0.9–8975.8)
Intercept	32.6 (42.2)				−4.8 (1.1) ***

Note: Reference level for school PA/sports without a coach and with a coach is “Never”; The dependent variables out-of-school PA/sports without a coach and with a coach have the following order: Never, <1/week, 1–3/week, ≥ 4 / week; PA = Physical Activity, MVPA = Moderate-to-Vigorous Physical Activity, PE = Physical Education; * *p* <0.05, ** *p* <0.01, *** *p* <0.001; betas (*b* = unstandardized and β = standardized) are presented for the linear regression (out-of-school leisure-time MVPA) and odds ratios are presented for the ordinal (out-of-school PA/sports, without a coach and with a coach) and binomial (PA guidelines met) regressions.
